# A PCR-RFLP method for genotyping of inversion 2R*c* in *Anopheles coluzzii*

**DOI:** 10.1186/s13071-021-04657-x

**Published:** 2021-03-22

**Authors:** Raquel Montanez‑Gonzalez, Alexandra C. Vallera, Maria Calzetta, Verena Pichler, Rachel R. Love, Moussa W. Guelbeogo, Roch K. Dabire, Marco Pombi, Carlo Costantini, Frederic Simard, Alessandra della Torre, Nora J. Besansky

**Affiliations:** 1 Department of Biological Sciences, University of Notre Dame, Notre Dame, IN 46556 USA; 2 Eck Institute for Global Health, University of Notre Dame, Notre Dame, IN 46556 USA; 3 Dipartimento di Sanità Pubblica e Malattie Infettive, Università “La Sapienza”, Istituto Pasteur-Fondazione Cenci-Bolognetti, 00185 Rome, Italy; 4 Centre National de Recherche et Formation sur Le Paludisme (CNRFP), Ouagadougou, Burkina Faso; 5 Institut de Recherche en Sciences de la Santé (IRSS)/Centre Muraz, Bobo-Dioulasso, Burkina Faso; 6 MIVEGEC, University of Montpellier, IRD, CNRS, Montpellier, France

**Keywords:** *Anopheles gambiae* complex, Chromosomal inversion, Inversion genotyping, Malaria vector, Molecular karyotyping, PCR-RFLP, Tag SNP

## Abstract

**Background:**

Genotyping of polymorphic chromosomal inversions in malaria vectors such as *An. coluzzii* Coetzee & Wilkerson is important, both because they cause cryptic population structure that can mislead vector analysis and control and because they influence epidemiologically relevant eco-phenotypes. The conventional cytogenetic method of genotyping is an impediment because it is labor intensive, requires specialized training, and can be applied only to one gender and developmental stage. Here, we circumvent these limitations by developing a simple and rapid molecular method of genotyping inversion 2R*c* in *An. coluzzii* that is both economical and field-friendly. This inversion is strongly implicated in temporal and spatial adaptations to climatic and ecological variation, particularly aridity.

**Methods:**

Using a set of tag single-nucleotide polymorphisms (SNPs) strongly correlated with inversion orientation, we identified those that overlapped restriction enzyme recognition sites and developed four polymerase chain reaction (PCR) restriction fragment length polymorphism (RFLP) assays that distinguish alternative allelic states at the tag SNPs. We assessed the performance of these assays using mosquito population samples from Burkina Faso that had been cytogenetically karyotyped as well as genotyped, using two complementary high-throughput molecular methods based on tag SNPs. Further validation was performed using mosquito population samples from additional West African (Benin, Mali, Senegal) and Central African (Cameroon) countries.

**Results:**

Of four assays tested, two were concordant with the 2R*c* cytogenetic karyotype > 90% of the time in all samples. We recommend that these two assays be employed in tandem for reliable genotyping. By accepting only those genotypic assignments where both assays agree, > 99% of assignments are expected to be accurate.

**Conclusions:**

We have developed tandem PCR-RFLP assays for the accurate genotyping of inversion 2R*c* in *An. coluzzii*. Because this approach is simple, inexpensive, and requires only basic molecular biology equipment, it is widely accessible. These provide a crucial tool for probing the molecular basis of eco-phenotypes relevant to malaria epidemiology and vector control. 
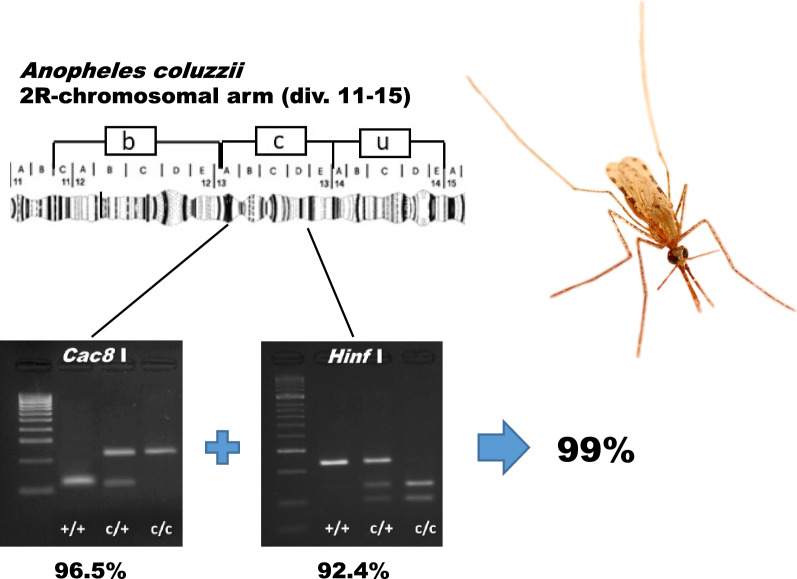

**Supplementary Information:**

The online version contains supplementary material available at 10.1186/s13071-021-04657-x.

## Background

Chromosomal inversions are taxonomically ubiquitous structural rearrangements resulting from the breakage and end-to-end reversal of a chromosome segment [1]. Growing numbers of studies powered by genomic sequencing suggest that inversions play important roles in sex chromosome evolution, speciation, and environmental adaptation, primarily because they suppress recombination between the inverted and corresponding non-inverted regions in heterozygotes [1–3]. Adaptive allelic combinations between inversion breakpoints are preserved as haplotype blocks against recombination with other genetic backgrounds. Balancing selection acting on environmentally adaptive variation appears to be a major force responsible for the maintenance of inversions and their involvement in local adaptation [1–4]. In most cases, however, the specific targets of selection within inversions and underlying molecular mechanisms controlling alternative phenotypes remain unknown.

The Afrotropical *Anopheles gambiae* complex radiated within the last 0.5 million years [5] into at least nine morphologically identical species [6–9], three of which are major vectors of human malaria. Across the complex, more than 120 inversion polymorphisms have been detected in natural populations, and an additional ten inversions are fixed between species [10]. Of note, the central part of chromosome 2R that overlaps the 2R*c* inversion in three taxa [*An. gambiae* (*s.s.*) (henceforth, *An. gambiae*), *An. coluzzii*, and *An. arabiensis*] is disproportionately involved in both fixed and polymorphic inversions in the species complex, potentially implicating the 2R*c* region in oviposition site specializations that distinguish taxa in this group [10]. Historically, fixed inversions provided crucial taxonomic tools for identification of these isomorphic species, while analysis of inversion polymorphisms such as 2R*c* segregating nonrandomly within species led to the recognition of assortatively mating ‘chromosomal forms’ presumed to be in the incipient stages of ecological speciation. One of these, the MOPTI chromosomal form (previously considered an incipient species of *An. gambiae* but now regarded as an arid-associated ecotype of *An. coluzzii* [6, 10]), was characterized in Mali by three main alternative whole-arm karyotypic arrangements: 2R*bc*, 2R*u*, and 2R + [11]. Importantly, the *bc* karyotypic arrangement (i.e. co-segregation of the adjacent 2R*b* and 2R*c* inversions on one arm) was found to be significantly correlated with climatic and ecological variation [11], as *bc* regularly increased in frequency both in the dry season and in dry geographic localities (Sahelian and Saharan). Mosquito carriers of the *bc* arrangement escape rain dependence through successful exploitation of irrigated sites for larval development and adult tolerance of extreme aridity. In an extensive review of these findings, the authors conclude: “The evidence for consistent temporal and spatial adaptive changes in (2R*bc*) inversion frequencies is unquestionable” [p.505, ref. 11].

Until the advent of molecular approaches, cytotaxonomy was the only practical method for distinguishing species of *An. gambiae* (*s.l.*), ensuring the preservation of cytogenetic karyotyping skills despite the drawbacks. Cytogenetic karyotyping is labor-intensive, requires specialized training, and is applicable only to properly preserved ovarian tissue from adult *An. gambiae* (*s.l.*) females at the half-gravid gonotrophic stage. The development of an rDNA-based PCR diagnostic assay beginning in 1993 [12] eliminated the requirement for cytotaxonomy and led to declining cytogenetic expertise, especially after the rDNA assay was extended to *An. coluzzii* (formerly M-molecular form, including MOPTI karyotypic arrangements) and *An. gambiae* (formerly S-molecular form, including mainly SAVANNA arrangements) [13].

Cytogenetic karyotyping had another important application in addition to species identification. Until recently, it was the only available tool for genotyping of inversion polymorphisms known to be significant predictors of epidemiologically relevant eco-phenotypes in *An. gambiae* (*s.l.*). As inversion polymorphism is a form of cryptic population substructure, unrecognized heterogeneities in population samples can bias genome-wide association studies as well as vector surveillance and control [14]. To address the need for an inversion genotyping method that does not rely on cytogenetics, we have been developing genomic approaches to identify and detect tag single nucleotide polymorphisms (SNPs) that are highly predictive of inversion orientation [15–17]. In addition to high-throughput molecular and *in silico* methods that can comprehensively genotype multiple tags from multiple inversions in parallel [15–17], we have also designed more conventional PCR-RFLP assays for individual inversions that, because they do not require expensive equipment or services, are both field- and budget-friendly [18]. Here, we complement our recently developed PCR-RFLP assays for inversion 2R*b* in *An. gambiae* and *An. coluzzii* [18] with assays for 2R*c* in *An. coluzzii*.

## Methods

### Mosquito sampling and processing

*Anopheles coluzzii* mosquitoes used in this study were from various historical Afrotropical collections motivated by polytene chromosome analysis of *An. gambiae* (*s.l.*). We focused our primary effort on an *An. coluzzii* population sampled from Burkina Faso, which lies in the arid Sudan savanna belt of West Africa. This geographic region was chosen because local *An. coluzzii* populations are characterized by high 2R*c* chromosomal inversion polymorphism [19]. Furthermore, the particular Burkina Faso population sample analyzed in this study (*n* = 463; Additional file 1: Table S1) had been previously genotyped for chromosomal inversions using each of three independent methodologies that collectively provide a robust basis for evaluating performance of individual PCR-RFLP assays developed here: (i) classical cytogenetics based on phase contrast microscopy and two molecular approaches, (ii) amplicon sequencing (GT-Seq) and (iii) array hybridization (TaqMan Open Array), based on the detection of tag single nucleotide polymorphisms (SNPs) strongly correlated with inversion orientation [15].

Additional geographic population samples of cytogenetically karyotyped *An. coluzzii* were obtained from three West African countries (Mali, *n* = 131 [11]; Senegal, *n* = 2 [20]; Benin, *n* = 20 [21]) and one Central African country (Cameroon, *n* = 43 [22]) (Additional file 1: Table S1).

*Molecular identification to species* DNA individually extracted by one of various methods was used as template in a PCR reaction for *An. coluzzii* species identification, based on the SINE200 assay [23] or ribosomal DNA (rDNA) [24, 25].

*Inversion genotyping *via* cytogenetics and multilocus tags* Polytene chromosome preparations from preserved ovarian nurse cells followed della Torre [26]. Paracentric inversion karyotypes were scored according to the *An. gambiae* cytogenetic map [10, 27, 28] and established nomenclature [11]. High-throughput molecular inversion genotyping based on the simultaneous detection of multiple tag SNPs was conducted previously via amplicon sequencing or probe hybridization to arrays, as detailed in Love et al. [15].

### Assay design for PCR-RFLP genotyping of *An. coluzzii* 2R*c*

Tag SNPs predictive of 2R*c* genotype were computationally identified previously [15, 16] in the Ag1000G database of natural genomic variation [29, 30], a database constructed from deeply sequenced wild-caught individual *An. gambiae* and *An. coluzzii* mosquitoes. At the time of our tag SNP ascertainment, population samples of *An. coluzzii* represented in Ag1000G came from Angola, Burkina Faso, Cameroon, Cote d’Ivoire, Ghana, Guinea, and Mali (we omitted samples from The Gambia and Guinea Bissau that were admixed with *An. gambiae*), and only a subset of those from Burkina Faso, Cameroon, and Mali carried metadata about cytogenetic karyotype. Accordingly, tag SNPs for *An. coluzzii* 2R*c* were identified in the pooled Burkina Faso and Angola Ag1000G samples following Ma and Amos [31], who showed that the application of principal components analysis (PCA) to SNP genotypes within the local window of the genome containing an inverted region produces a pattern indicative of two distinct “populations” of inversion homozygotes (inverted and standard) and their 1:1 admixture (inversion heterozygotes), a pattern of population substructure created by suppressed recombination in the inverted region.

Briefly, to apply this approach, we created a matrix of one-digit genotypes at biallelic SNPs within 2R*c* for each mosquito. One-digit SNP genotypes represent the count (0, 1, or 2) of alternate alleles (i.e. variants with respect to the PEST reference sequence at a SNP position). PCA of the resulting data matrix allowed computational imputation of the 2R*c* inversion genotype for mosquitoes in the sample. Individual SNPs capable of accurately predicting inversion genotype (tag SNPs) should have allelic states that are strongly correlated with inversion genotype. The correlation at individual candidate tags was measured as the percentage of the total mosquito sample with matching inversion and SNP genotypes. To be conservative, we calculated the correlation separately for each of the three inversion genotypes, adopting the minimum value across the three genotypes as the final genotypic concordance value. Candidate tags were ranked based on the final genotypic concordance values.

Unlike high-throughput molecular inversion genotyping based on tens of tags per inversion, conventional PCR-RFLP genotyping necessarily relies on one or a few tags, each of which should thus have the highest genotypic concordance. Although we prioritized such tags, suitable candidates for RFLP also depend on the serendipitous overlap of the tag SNP with the recognition site of a commercially available restriction enzyme, such that recognition and cleavage depend upon allelic status of the tag on both chromosomes in a diploid mosquito. Additional constraints include the ability to design suitable flanking PCR primers (e.g. free of possible hairpin or primer-primer interactions), which anneal to high-complexity, non-repetitive template DNA to reduce off-target mis-priming. Applying a minimum threshold of 85% genotypic concordance to the ranked tags, we screened the qualified tags for those that overlapped restriction enzyme recognition sites, using NEBcutter v2.0 [32]. Using the *An. gambiae* PEST reference genome accessed through VectorBase [33] and Primer3Plus v2.4.2 [34], we designed primer pairs that flanked each tag SNP and produced amplicons 200–300 bp in length. We avoided any primer binding sites and restriction enzyme recognition sites that contained high frequency variants (> 5%, as judged from Ag1000G variation data) and also excluded primer binding sites that overlapped repetitive sequence (as judged from softmasking of AgamP4). Assays whose electrophoretic profiles provided the best separation between inversion genotypes were prioritized.

### PCR-RFLP genotyping

PCR was carried out in 25 µl reactions containing 20 mM Tris–HCl (pH 8.3), 50 mM KCl, 200 µM of each dNTP, 2 mM MgCl_2_, 10 pmol of each primer, 1 U of Taq polymerase, and 1 µl of template genomic DNA. PCR conditions included an initial incubation at 94 °C for 2 min, 35 cycles of 94 °C for 30 s, 57 °C for 30 s, and 72 °C for 45 s, followed by 72 °C for 2 min and a 4 °C hold. PCR amplification was confirmed via gel electrophoresis on 1% agarose gels stained with SYBR Safe at 135 V in 0.5 × TBE buffer.

An 8 µl aliquot of the resulting PCR product was digested in 20 µl reactions with 0.5 µl restriction enzyme and 1 × Cutsmart buffer following the recommendations of the manufacturer (New England Biolabs, Ipswich, MA, USA): HinfI and HaeII reactions were incubated at 37 °C for 1 h, then heat inactivated at 80 °C for 20 min; Cac8I incubated at 37 °C for 1 h, then heat inactivated at 65 °C for 20 min; BstUI incubated at 60 °C for 1 h without heat inactivation. Optionally, SDS loading dye was prepared with 10 µl of 10% SDS and 1 ml of 6 × loading dye to mitigate protein-DNA interactions and improve band quality. Digest products were analyzed via gel electrophoresis on 2.5–3% agarose gels stained with SYBR Safe at 100 V in 0.5 × TBE buffer.

### Amplicon sequencing

Enzymatic cleanup of the amplified PCR product was achieved in reactions containing 2 U of Exonuclease 1 (USB Corporation, Cleveland, OH), 1U of Shrimp Alkaline Phosphate (USB), 1.8 µl of ddH_2_O, and 8 µl of the PCR product. After incubation at 37 °C for 15 min, the enzymes were inactivated at 80 °C for 15 min. Sanger sequencing was performed directly on the resulting samples, using the forward PCR primer (Table 1) and the ABI 3730X1 DNA Analyzer Platform (ThermoFisher Scientific, Waltham, MA), by the Genomics and Bioinformatics Core Facility at the University of Notre Dame or by Eurofins Genomics Italy. Sequences were deposited with GenBank under accessions MW158473-MW158543.

**Table 1:** PCR-RFLP genotyping assays for inversion 2R*c* in *An. coluzzii*

Tag position (breakpoints)	Concord	Ref/Alt	RE	Chr cut	Primer Pairs (5′➝3′)	Amplicon (bp)	Cleavage products (bp)
(2R:2675000)							
2R:27283425	89.5%	G/A	Cac8I	2R+^c^	F: TAATCGTGTTCGTTCGGTCA R: CCGGGTACACCTGGAAAGTA	239	125, 114
2R:28121178	87.2%	C/T	BstUI	2R +^c^	F: AAATGCGTGTTGCACTTGAC R: CCAATAAATCCTAACCCACACG	224	173, 51
2R:30386671^a^	86.9%	G/A	HaeII	2R +^c^	F: GGAAAGTTCACGAGCCAAAA R: TGAGCTTTAGCGACTGCAAG	270	165, 105
2R:30392408^b^	94.1%	A/G	HinfI	2Rc	F: AGCTGCCAGGATTTTGTACG R: CGGCGGGGAAAGTAATTTAT	233	136, 97
(2R:31473100)							

Tag position (breakpoints): chromosome coordinates of tag SNP and estimated breakpoint positions from Sangaré [35]; Concord: minimum percent concordance of tag with inversion genotype in Ag1000G based on Love et al. [15]; Ref/Alt, reference and alternate allele at tag SNP; RE, restriction enzyme; chr cut, chromosome (inverted or standard) expected to be cleaved in the assay

^a^Tag SNP also employed in both high-throughput genotyping panels as described in Love et al. [15]

^b^Tag SNP also employed in the amplicon sequencing genotyping panel as described in Love et al. [15]

## Results and discussion

**Fig. 1:**
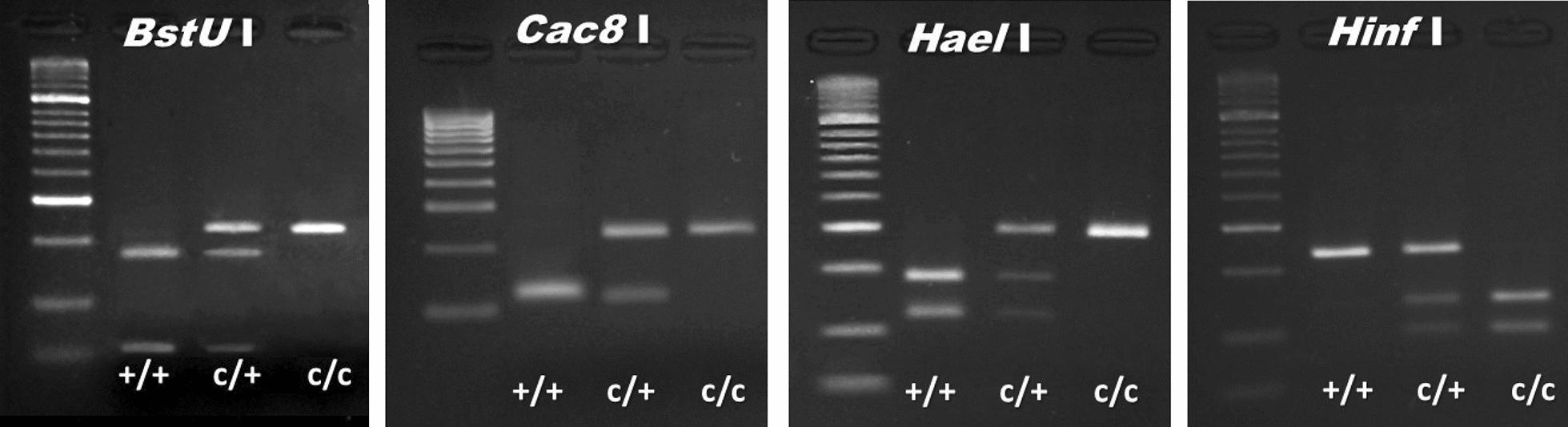
Representative electrophoretic profiles of the BstUI, Cac8I, HaeII, and HinfI assays for inversion genotyping of 2R*c*. Standard (uninverted) homozygotes for 2R*c*, + / +; heterozygotes, c/ +; inverted homozyogtes, c/c. Molecular weight markers (Bioline; Memphis, TN, USA) are the 50-bp ladder for BstUI, HaeII, and HinfI assays and the 100-bp ladder for Cac8I

Four candidate tag SNPs met the four design criteria for PCR-RFLP assays (see”Methods”): (i) ≥ 85% genotypic concordance in the Ag1000G database; (ii) overlap with a restriction enzyme recognition site; (iii) PCR primers fulfilling Primer3Plus default parameters; (iv) clearly distinguishable electrophoretic profiles among inversion genotypes (Table 1, Fig. 1). For simplicity, we named these assays according to the restriction enzyme employed: Cac8I, BstUI, HaeII, and HinfI.

Note that owing to distinct filtering requirements among molecular approaches, two of the four tag SNPs targeted by these assays differ from those employed in the respective sets of 23 and 11 tags developed for high-throughput amplicon sequencing and array hybridization genotyping of 2R*c* [15] (see Table 1), though the common principles underlying all 2R*c* tags suggest that their performance should be similar.

### Validation in the Burkina Faso sample

We previously genotyped 2R*c* in a sample of 463 *An. coluzzii* collected from Burkina Faso, using three independent methods: cytogenetic karyotyping, high-throughput amplicon sequencing, and TaqMan array hybridization (see Methods). Three-way concordance among the methods exceeded 94%, and two-way agreement between the molecular approaches was even higher (97.7%) despite the fact that only two tag SNPs were shared between them [15]. Genomic DNA remaining from specimens subjected to this three-fold genotyping was employed in the validation of the four PCR-RFLP assays. We defined the reference 2R*c* genotype for each specimen—the ‘gold standard’ against which PCR-RFLP assays were compared—as the consensus genotype indicated by at least two of the three previous methods (Additional file 1: Table S1). Aggregate and individual PCR-RFLP assay genotypes were compared against this reference 2R*c* genotype. The aggregate PCR-RFLP assay genotype was defined by majority rule, i.e. at least three of four PCR-RFLP assays agreed. There were 26 of 463 specimens (5.6%) for which no aggregate genotype could be defined, either because two different genotypes were each supported by two assays or three different genotypes were supported among the four (Additional file 1: Table S1). Of the remaining 437 specimens that could be assigned aggregate genotypes, all matched the corresponding reference 2R*c* genotype (Table 2; Additional file 1: Table S1).

**Table 2:** Concordance between the reference 2R*c* inversion genotype and PCR-RFLP assay genotypes in the Burkina Faso sample

REF	AGG			Cac8I			BstUI			HaeII			HinfI		
	0	1	2	0	1	2	0	1	2	0	1	2	0	1	2
0	*143*	0	0	*145*	3	0	*116*	28	4	**130**	16	2	*144*	3	1
1	0	*214*	0	4	*218*	7	17	*189*	23	15	*199*	15	16	*211*	2
2	0	0	*80*	0	2	*84*	0	19	*67*	0	5	*81*	1	12	*73*
Concord (%)	437/437 (100%)			447/463 (96.5%)			372/463 (80.3%)			410/463 (88.6%)			428/463 (92.4%)		

Italic values are those concordant between cytogenetics and the assay

REF, reference genotype (0, standard homozygote; 1, heterozygote; 2, inverted homozygote); AGG, aggregate PCR-RFLP genotype (see text); concord (%): concordance between reference and assay 2R*c* genotypes

Not surprisingly, the concordance between individual PCR-RFLP assays and the reference 2R*c* genotype was imperfect, varying from ~ 80% to ~ 97% (Table 2). The most important factor underlying incomplete congruence is simply that none of the tag SNPs detected by PCR-RFLP assays are deterministic for inversion orientation in the Ag1000G variation database, presumably owing to low levels of recombination and gene conversion between opposite orientations in heterozygotes (see Ref. 36). As such, no single assay will unerringly predict the correct inversion genotype. Moreover, the percent concordance between tag and chromosomal arrangement observed in different population samples is expected to vary at least somewhat from the value observed in the Ag1000G database, for stochastic reasons alone, if not due to temporal, geographic, or other population genetic differences. Additional (non-exclusive) more minor sources of disagreement between the reference genotype and the genotype predicted by PCR-RFLP assays include SNP variation in the enzyme recognition site at positions other than the tag itself, SNP variation in the primer binding sites on one or both chromosomal arrangements that reduce or preclude primer binding (often referred to as ‘allelic dropout’ and typically recognized as a heterozygote deficit), and technical problems with restriction digestion (partial or complete failure of the restriction enzyme to cleave an intact target site).

The two assays that were least concordant with the reference genotype in the Burkina Faso sample were BstUI (80.3%) and HaeII (88.6%). In neither case did we find evidence for significant heterozygote deficits or any striking imbalance in the distribution of discordances across genotypes. In fact, the HaeII assay in our sample slightly outperformed its predicted genotypic concordance (87% based on the Ag1000G database; Table 1), suggesting that this factor alone is sufficient to explain the HaeII assay’s performance. The BstUI assay, by contrast, underperformed its predicted genotypic concordance in Ag1000G (87%; Table 1). We sequenced a subset of 19 BstUI amplicons from specimens whose PCR-RFLP assay disagreed with the reference genotype (Additional file 1: Table S1). We identified three cases in which an additional SNP destroyed the BstUI restriction target site and abrogated cleavage (despite the allelic state of the tag SNP matching the reference genotype in all three cases), which at least partly explains the apparent underperformance of this assay.

The remaining two assays, Cac8I and HinfI, agreed more often with the reference genotype (96.5% and 92.4%, respectively; Table 2). The Cac8I assay performed considerably better in the Burkina Faso sample than predicted based on the genotypic concordance of the tag in Ag1000G (89.5%; Table 1). Of the 16 specimens with discrepant genotypes, we sequenced the Cac8I amplicons of 12 (Additional file 1: Table S1), finding three whose discrepancies were explained not by the allelic state of the tag SNP (which agreed with the reference genotype in all three specimens) but by a different SNP that destroyed the restriction target site. The performance of HinfI closely matched expectations based on its tag in Ag1000G (94.1%; Table 1); we did not perform sequencing on HinfI amplicons from Burkina Faso owing to COVID-19 restrictions, but we have some insight based on sequencing data from other population samples (see below).

### Validation in other population samples

To extend our analysis spatially, we analyzed samples from four additional countries in West and Central Africa (Mali, Senegal, Benin, Cameroon) that had been subject to cytogenetic analysis, but not high-throughput molecular genotyping approaches. For these specimens, the reference 2R*c* genotype was based solely on cytogenetics, although this method is not without human error (up to 4% or more, depending upon degree of training and experience; [16, 18]). We compared the cytogenetic genotype to the aggregate (majority rule) and individual PCR-RFLP assay genotypes in pooled (Table 3) and individual (Additional file 2: Table S2) population samples. Due to small sample size and lack of 2R*c* inversion polymorphism in some individual population samples, we present below the results based on pooled samples. Similar to the Burkina Faso population sample, we found nearly perfect concordance (98%) between cytogenetics and the aggregate genotype (184 of 187), after excluding nine specimens whose four PCR-RFLP assays did not produce a majority genotype. Among the three discrepancies (Table 3), one specimen showed a ‘0′ karyotype (i.e. homozygous standard, confirmed cytogenetically) contradicted by three of four PCR-RFLP assays showing a genotype of ‘1′ (i.e. heterozygote, with the *BstU*I gentotypes confirmed by sequencing). The other two specimens showed a ‘1′ karyotype, contradicted by all four PCR-RFLP assays indicating a ‘0′ genotype. In both specimens, an inversion loop in the 2R*c* region was confirmed cytogenetically but, due to the relatively low quality of the polytene chromosomes, it was not possible to rule out that the loop corresponds to a rare inversion in the same chromosomal region [28].

**Table 3:** Concordance between the cytogenetic 2R*c* inversion genotype and PCR-RFLP assay genotypes in pooled samples from Senegal, Mali, Benin, and Cameroon

CYT	AGG			Cac8I			BstUI			HaeII			HinfI		
	0	1	2	0	1	2	0	1	2	0	1	2	0	1	2
0	*88*	1	**0**	*86*	6	2	*82*	9	3	*70*	22	2	*91*	3	0
1	2	*62*		4	*58*	3	5	*59*	1	4	*57*	4	3	*62*	0
2	0	0	*34*	0	2	*35*	2	3	*32*	0	4	*33*	0	8	*29*
Concord (%)	184/187 (98.4%)			179/196 (91.3%)			173/196 (88.3%)			160/196 (81.6%)			182/196 (92.9%)		

Italic values are those concordant between cytogenetics and the assay

CYT, cytogenetic genotype (0, standard homozygote; 1, heterozygote; 2, inverted homozygote); AGG, aggregate PCR-RFLP genotype (see text); concord (%), concordance between CYT and assay 2R*c* genotypes

Qualitatively, the performance of individual PCR-RFLP assays was comparable inside and outside of Burkina Faso. The same two assays with concordances < 90% in Burkina Faso (BstUI and HaeII) also performed below 90% elsewhere (Table 3). Considering that the concordance of their tag SNPs was ~ 87% in Ag1000G, these assays actually met expectations. However, the higher correlation between the tag SNPs of the other two assays (Cac8I and HinfI) and inversion status in Ag1000G make them better prospective candidates than BstUI and HaeII. Indeed, in agreement with results from Burkina Faso, both Cac8I and HinfI were superior at genotyping elsewhere in West and Central Africa. Although the Cac8I assay in these other samples did not match its 96.5% performance in Burkina Faso, it was nevertheless concordant with cytogenetics > 91% of the time. Sequencing the PCR amplicons of a subset of nine specimens with discordances between the Cac8I assay and cytogenetics revealed that in five cases the tag SNP genotype actually agreed with cytogenetics. In two of those cases, the PCR-RFLP assay disagreement was caused by a different SNP that destroyed the restriction site. Similarly, the HinfI assay was ~ 93% concordant with cytogenetics in these same samples. Sequencing of 11 PCR amplicons from specimens with genotypic discordances between the assay and cytogenetics revealed no additional polymorphisms in the HinfI recognition site.

Our previous efforts to develop tag SNPs for inversion genotyping were directed toward maximizing geographic and taxonomic inclusion based on *An. coluzzii* and *An. gambiae* samples represented in Ag1000G at the time [16]. For three inversions (2L*a*, 2R*b*, and 2R*u*), a single set of tags was identified that successfully genotyped both sister species [15, 16]. By contrast, population structure between *An. coluzzii* and *An. gambiae* in the 2R*j*, 2R*d*, and 2R*c* arrangements dictated the development of taxon-specific tags [15, 16]. For 2R*j* and 2R*d*, the tags are specific for *An. gambiae* and are not applicable in *An. coluzzii*. In the case of inversion 2R*c*, separate tag sets were successfully developed for *in silico* genotyping of both taxa [16]. However, application of these tags for high-throughput molecular genotyping of Burkina Faso samples (independent of Ag1000G) revealed that only *An. coluzzii* tags performed faithfully against cytogenetically karyotyped *An. coluzzii* specimens; *An. gambiae* tags applied to *An. gambiae* specimens were inadequate [15]. Hence, in the present work, using these same Burkina Faso samples (and others), we focused our PCR-RFLP assay development exclusively on *An. coluzzii*. The reasons for heterogeneous tag performance across different taxa and even among population samples of the same taxon have yet to be examined in detail, but are likely caused by population structure, which uncouples the correlation between a tag SNP and inversion orientation. Possible (non-exclusive) sources of population structure, aside from taxonomic boundaries themselves, include geography, different selective regimes on allelic targets within inversions, and/or different molecular origins of the inversion. With respect to the last factor, indirect evidence based on allelic variation near the 2R*c* breakpoints is consistent with the idea of multiple origins [16], although computational haplotype phasing or long molecule sequencing leading to molecular breakpoint characterization will be required for a confident resolution. Inversion 2R*c* is also peculiar in that it is almost never found alone on chromosome 2R. Instead, 2R*c* is in nearly perfect linkage disequilibrium with the inverted arrangement of either 2R*b* (i.e. 2R*bc*) or 2R*u* (i.e. 2R*cu*) [10, 16]. Of note, *cu* is a characteristic SAVANNA karyotypic arrangement common in many populations of *An. gambiae* (*s.s.*), while *bc* is a characteristic MOPTI karyotypic arrangement that predominates in arid populations of *An. coluzzii*. Except for a highly endemic chromosomal form of *An. gambiae* (*s.s.*) known as BAMAKO [10, 11], *An. gambiae* carriers of the *cu* arrangement are underrepresented in the Ag1000G database. Future development of 2R*c* tags and genotyping assays in non-BAMAKO *An. gambiae* may benefit from additional whole-genome sequencing of *An. gambiae* carriers of *cu*.

Several factors, most importantly the non-deterministic nature of tag SNPs predictive of 2R*c* genotype, operate to prevent any single tag—and any single PCR-RFLP assay dependent on that tag—from unerringly predicting the correct inversion genotype. However, we have shown here that the joint application of multiple PCR-RFLP assays targeting different tags can substantially improve genotypic concordance. For laboratories unwilling or unable to invest in high-throughput genotyping, the most efficient strategy for accurate genotyping of 2R*c* which minimizes ‘false positives’ at the cost of some ‘false negatives,’ would be to apply both the Cac8I and HinfI assays jointly to each specimen in a population sample, preserving only those specimens with genotypes supported by both assays. Had this approach been adopted in the Burkina Faso sample of 463 *An. coluzzii*, 414 specimens (89.4%) would have had concordant Cac8I and HinfI assay genotypes. Of those 414, all genotypes except one (99.8%) would have agreed with the reference 2R*c* genotype. From the original sample of 463, 49 specimens (10.6%) would have been excluded because of conflicting PCR-RFLP assay genotypes.

There are several limitations of any PCR-RFLP approach. First, and arguably most important, this method is premised on a naturally occurring restriction site overlapping the tag SNP of interest, severely limiting the choice of amenable tag SNPs. Second, the process requires two steps, hence more time: PCR, followed by restriction digestion. Third, restriction enzymes may be costly, difficult to obtain commercially, and labile even if handled carefully. Finally, genotyping errors result both from technical failures of restriction digestion even if the cut site is intact, and from additional polymorphisms arising in the restriction enzyme recognition sequence that destroy the site. We recommend the consistent use of positive controls as indicators of successful restriction enzyme activity. However, beyond this best practice, the other limitations remain. Overcoming these limitations requires the development of a genotyping assay that dispenses with the need for restriction digestion of the amplicon. Quite recently, a rapid and inexpensive approach termed “SuperSelective (SS) PCR” was developed to genotype single nucleotide variants in *Caenorhabditis elegans* directly following endpoint PCR [37]. This approach has broad application in any genetic system including *An. coluzzii.* Moreover, it can be developed for any tag, irrespective of overlap with a restriction enzyme target site, as it avoids the RFLP step entirely and depends only on specific PCR detection of the SNP itself. Accordingly, to overcome the limitations of PCR-RFLP for inversion genotyping, future efforts should be directed at optimizing this SS approach, focusing on those tag SNPs with the highest levels of genotypic concordance in Ag1000G.

## Conclusions

Although improved technological approaches can be anticipated in the future, we have developed two serviceable PCR-RFLP assays, Cac8I and HinfI, that, when used in tandem, allow highly accurate genotyping of inversion 2R*c* in *An. coluzzii* without the need for specialized equipment or training. Because they are rapid and inexpensive, they can be applied in field settings. These assays provide invaluable tools to investigate the association between 2R*c* and ecologically or epidemiologically relevant phenotypes and advance our understanding of their molecular basis, aiding our understanding of the evolutionary diversification of this important malaria vector.

## Supplementary Information

**Additional file 1. Table S1.** Total sample metadata and inversion genotypes for 2R*c*

**Additional file 2. Table S2.** Concordance between the cytogenetic 2R*c* inversion genotype and PCR-RFLP assay genotypes in samples from Senegal, Mali, Benin, and Cameroon.

## Data Availability

Data supporting the conclusions of this article are included in the article and its supplementary files. Sequences determined from this study are available in GenBank under accession numbers MW158473-MW158543.
