# Supplementary material for: A PCR-RFLP method for genotyping of inversion 2Rc in Anopheles coluzzii
Source: Parasit Vectors. 2021 Mar 22;14:174. doi: 10.1186/s13071-021-04657-x (PMC7983089; doi:10.1186/s13071-021-04657-x)
Supplement: Supplementary file 2 — Additional file 2. Table S2. Concordance between the cytogenetic 2Rc inversion genotype and PCR-RFLP assay genotypes in samples from Senegal, Mali, Benin, and Cameroon. [file 13071_2021_4657_MOESM2_ESM.docx]

Table S2. Concordance between the cytogenetic 2R*c* inversion genotype and PCR-RFLP assay genotypes in samples from Senegal, Mali, Benin, and Cameroon.

| Country | CYT | Cac8I | | | BstUI | | | HaeII | | | HinfI | | |
| --- | --- | --- | --- | --- | --- | --- | --- | --- | --- | --- | --- | --- | --- |
|  |  | 0 | 1 | 2 | 0 | 1 | 2 | 0 | 1 | 2 | 0 | 1 | 2 |
| Mali | 0 | **23** | 4 | 2 | **27** | 1 | 1 | **26** | 3 | 0 | **29** | 0 | 0 |
|  | 1 | 4 | **58** | 3 | 5 | **59** | 1 | 4 | **57** | 4 | 3 | **62** | 0 |
|  | 2 | 0 | 2 | **35** | 2 | 3 | **32** | 0 | 4 | **33** | 0 | 8 | **29** |
| Senegal | 0 | **2** | 0 | 0 | **1** | 1 | 0 | **1** | 1 | 0 | **2** | 0 | 0 |
|  | 1 | 0 | **0** | 0 | 0 | **0** | 0 | 0 | **0** | 0 | 0 | 0 | 0 |
|  | 2 | 0 | 0 | **0** | 0 | 0 | **0** | 0 | 0 | **0** | 0 | 0 | 0 |
| Benin | 0 | **18** | 2 | 0 | **13** | 6 | 1 | **20** | 0 | 0 | **20** | 0 | 0 |
|  | 1 | 0 | **0** | 0 | 0 | **0** | 0 | 0 | **0** | 0 | 0 | **0** | 0 |
|  | 2 | 0 | 0 | **0** | 0 | 0 | **0** | 0 | 0 | **0** | 0 | 0 | **0** |
| Cameroon | 0 | **43** | 0 | 0 | **41** | 1 | 1 | **23** | 18 | 2 | **40** | 3 | 0 |
|  | 1 | 0 | **0** | 0 | 0 | **0** | 0 | 0 | **0** | 0 | 0 | **0** | 0 |
|  | 2 | 0 | 0 | **0** | 0 | 0 | **0** | 0 | 0 | **0** | 0 | 0 | **0** |
|  | Concord  (%) | 179/196  (91.3%) | | | 173/196  (88.3%) | | | 160/196  (81.6%) | | | 182/196  (92.9%) | | |
